# Demographic and temporal trends in mental health and substance use services provided by primary care physicians in British Columbia, Canada

**DOI:** 10.1186/s12875-024-02587-y

**Published:** 2024-09-10

**Authors:** Margaret Jamieson, Myriam Juda, M. Ruth Lavergne, Paul Kurdyak, Audrey Laporte, David Rudoler

**Affiliations:** 1 Institute of Health Policy, Management and Evaluation, University of Toronto, 155 College Street, Suite 425, Toronto, ON M5T 3M6 Canada; 2 Department of Psychology, Simon Fraser University, Robert C. Brown Hall 5246, 8888 University Drive, Burnaby, V5A 1S6 Canada; 3 Telfer School of Management, University of Ottawa, 5105-55 Laurier Avenue East, Ottawa, K1N 6N5 Canada; 4 Department of Family Medicine, Dalhousie University, 5909 Veterans Memorial Lane, Halifax, B3H 2E2 Canada; 5 Institute for Mental Health Policy Research, Centre for Addiction and Mental Health (CAMH), 250 College Street, Toronto, M5T 1R8 Canada; 6 ICES, Toronto, Canada; 7 Department of Psychiatry, University of Toronto, 250 College Street, 8th Floor, Toronto, M5T 1R8 Canada; 8 Faculty of Health Sciences, University of Ontario Institute of Technology, 2000 Simcoe Street North, Oshawa, L1H 7K4 Canada; 9 Ontario Shores Centre for Mental Health Sciences, 700 Gordon Street, Whitby, L1N 5S9 Canada; 10 Canadian Centre for Health Economics, 155 College Street, Suite 425, Toronto, M5T 3M6 Canada

**Keywords:** Practice patterns, Physicians’, Mental health, Substance-related disorders, Canada, British Columbia

## Abstract

**Background:**

As the demand for mental health and substance use (MHSU) services increases, there will be an even greater need for health human resources to deliver this care. This study investigates how family physicians’ (FP) contact volume, and more specifically, MHSU contact volume, is shaped by demographic trends among FPs in British Columbia, Canada.

**Methods:**

We used annual physician-level administrative billing data and demographic information on FPs in British Columbia between 1996 and 2017. This study analyzes trends in primary care service provision among graduating cohorts of FPs, FPs of different ages (as measured by years since graduation), and FPs practicing during different time periods. Additionally, analyses are stratified by FP sex to account for potential differences in labour supply patterns between male and female FPs.

**Results:**

Our results show that while FPs’ overall contacts with patients decreased between 1996 and 2017, their annual number of MHSU contacts increased, which was largely driven by an increase in substance use visits. Demographically, the proportion of female FPs in the labour force rose over time. Observed trends were similar, though not identical in male and female FPs, as males tended to have higher overall contact volume (both total contacts and MHSU), but also steeper declines in contact volume in later careers. The number of contacts (both total and MHSU) changed across career stage - rising steadily from start to mid-career, peaking at 20–30 years in practice, and decreasing steadily thereafter. This was evident for all cohorts and consistent over the 21-year study period but flattened in amplitude over time. Our findings also point to potential cohort effects on labour supply. The inverse U-shaped career trend extended to MHSU contacts, but its peak seems to have shifted to a later career stage (peaking at 30–40 years of practice) over time.

**Conclusions:**

Our study shows changing dynamics in MHSU service delivery among FPs over time, across the life span and between FP sexes that are likely to influence access to care beyond simply the number of FPs. Given the healthcare needs of the population, these findings point to potential future changes in provision of MHSU services.

**Supplementary Information:**

The online version contains supplementary material available at 10.1186/s12875-024-02587-y.

## Background

Canada is currently facing a double crisis: increasing rates of mental illness and opioid overdose [1] and a shortage in access to primary care [2–5]. This is despite the growing per capita supply of family physicians (FPs) over the last 30 years [6]. Among the most affected by this crisis was the province of British Columbia (BC) [7, 8], which has nearly double the national age-adjusted rate of opioid toxicity deaths [1]. Changes in FPs’ demographics, such as cohort and age distributions, may affect the delivery of and access to mental health and substance use (MHSU) services in BC, as well as changing healthcare needs of the population – motivating our analysis. In the wake of the COVID-19 pandemic and its impact on both MHSU service demands and burnout among FPs [9, 10], an understanding on how physician-level demographics and practice patterns has contributed to changing access to primary MHSU services in BC is vital to understanding how best to address these problems in the future.

When compared to the rest of Canada, BC has a consistently higher level of unmet mental health care needs identified in population-level surveys [8, 11], and has nearly double the national age-adjusted rate of opioid toxicity deaths (33.9 compared with 17.2 per 100,000 in 2020) [1]. BC has the highest rates of hospitalization due to mental illness and substance use in the country and it is estimated that approximately 25% of the population will experience a substance use disorder at some point in their lives [12, 13]. Additionally, BC is currently under a state of emergency due to a toxic drug crisis [14]. There has been a spike in toxic-drug related deaths in BC beginning in 2016 due to a surge in the availability of substances tainted with fentanyl – a powerful opioid roughly 20–40 times more potent than heroin that has been causing overdoses in drug users [14, 15]. Although it is at the forefront of MHSU discussions at the moment, the spike in toxic-drug related deaths in BC did not occur until 2016 – at the very end of our observed time period [14]. There is evidence that leading up to 2016, engagement in treatment for opioid use disorder was trending upwards in the province – though that improvement stopped with the escalation of the opioid crisis [16].

Primary care plays a critical role in the delivery of MHSU care. Primary care providers, including FPs are often the first point of contact for patients with MHSU concerns, and they have a unique opportunity to identify and address these issues early on [17]. Most mild to moderate MHSU concerns are managed by FPs in BC [13]; however, the scope of their treatment is often limited by the extent of the training a FP has in providing MHSU-specific care [18–21]. administrative barriers (such as limits on the number of counselling sessions they can provide annually) [22], and time constraints on these FPs. A Quebec study found that among FPs from a variety of practice settings, at least 20% of their visits in 2011 were from patients with common mental disorders [20]. The FPs reported generally high levels of comfort in dealing with common mental disorders.

Patient contacts per FP, in general, have fallen in Canada [23–25], with much of the blame being placed on younger generations [26–28] However, studies have shown decreasing contact volume among physicians at all career stages [2, 23, 25, 29, 30], not just among those entering practice. This suggests that other demographic changes may be at play here. Research on Canadian physicians has shown that contact volume changes over a clinician’s career stage (inverse U-shape). The amount of patient contact increases during a physician’s early career, peaking at 27–29 years in practice, after which it decreases again until retirement [2, 25, 29, 31]. Evidence from Quebec suggests that younger generations of FPs were less likely to take on a long-term commitment with patients with complex MHSU disorders [19]. In addition to patients with complex MHSU disorders, patients with mild to moderate depression and anxiety are also experiencing changes in service access, with other evidence from 2010 suggests that Canadian FPs under the age of 44 and over the age of 65 were less likely to provide mental health counseling [32]. It is unclear, however, whether these findings reflect a cohort effect versus a career stage effect.

This study explores changing dynamics in MHSU service delivery among FPs over time, across the life span, and across male and female FPs that have influenced access to care between 1996 and 2017. According to empirical models, age interacts with sex, as women tend to reduce their workload between the ages of 30 and 45 years [4, 33, 34]. As such, this study accounts for FP sex. In line with the prevailing economic literature, we predict that MHSU visit volume will peak mid-career. The influence of the graduating cohort and time period on MHSU contact volume is unclear a priori. Our study is the first to report on this topic. A better understanding of the factors driving the shortage of access to MHSU services is urgently needed. This study has the potential to make an important contribution to policy concerning human health resource planning. Although it is not the only factor in ensuring that patient needs are met, understanding the trends in MHSU supply by FPs will inform training, recruitment, and retention efforts that could improve much-needed access to primary MHSU care.

## Methods

### Data sources and study population

This study used administrative data from Population Data BC, a centralized repository and secure environment for linking a variety of deidentified longitudinal health and social data sources in BC. The data holdings cover FP characteristics, service provision, and payment data for all insured medical services throughout the province’s publicly funded healthcare system. The datasets cover all registered FPs and all patient contacts for which the government is billed, and linkages between datasets are deterministically linked using personal identifiers. Data from all FPs with active practices in the province of BC between 1996 and 2017, with existing data on self-reported sex and graduation year, were included in this study. FPs with “active practices” were identified using a threshold of at least 100 patient contacts annually, to ensure our results were not skewed by FPs with very low service volumes who might have very different approaches to practice. We defined “family physicians” as those who had completed a family medicine residency and general practitioners who trained prior to mandatory residency, as described in the early 1990s.

We used the Medical Services Plan (MSP) Payment Information data file to identify FPs who had billed for services [35–37], including relevant International Classification Board 9 (ICD-9) codes (see Additional File 1), and the College of Physicians and Surgeons of BC (CPSBC) registry to determine FP demographic information, such as age, graduation year and place and practice specialty (e.g., family medicine family medicine). We used registration and billing patterns to identify eligible FPs. For each fiscal year, we identified FPs based on their specialty registration and billing patterns. Because the registration allows billing for multiple specialties, we examined the total costs for each specialty and classifying physicians according to their top-billed specialty. Registry information also included self-reported sex, which was recorded as a binary variable (male or female) at the time of registration. Any results that could identify populations with a sample size < 5 were excluded. This study received ethics board approval from the University of Toronto (RIS Human Protocol number 39788).

### Variables and analytic methods

Our outcomes of interest include overall service volume and MHSU service volume. Overall service volume is measured by the number of unique FP-patient-day contacts. This means that every visit by a patient is considered a contact regardless of how many different fee items were billed for the encounter. In cases where the same patient had multiple visits per day with the same physician, we only count this as one contact. MHSU service volume was measured by unique FP-patient-day combinations where one or more ICD-9 codes related to mental health or substance use were recorded in the billing record. The Steele algorithm was used to identify diagnostic codes that indicate the delivery of MHSU services, as shown in Table S1 in Additional File 1 [38].

Key explanatory variables include *years since graduation* (binned into ten-year-long categories) as a measure of career stage, *graduation year* from medical school (binned into ten-year-long categories) as a measure of generational cohort, [30, 39] and *time period* (binned into four-year-long categories to maximize sensitivity to period-level changes and interpretability). Our analysis also included self-reported FP *sex* at the time of registration (male, female) and *practice location* (urban, small urban, rural) based on the statistical area classification of the census subdivision [40, 41] of the residential address where most patient contacts took place for a given FP in a given year. In cases where there was a tie between two classifications, the more urban option was chosen.

### Analytic strategy

The analyses were conducted using the statistical software R (version 4.0.03) and involved the use of graphical analyses and mean comparisons. First, we summarized the total number of FPs stratified by *graduation cohort*, *career stage*, *sex*, and *time period*. Next, we summarized the mean number of overall and MHSU annual FP-patient-day contacts (henceforth known simply as “contacts”) across these stratifications and graphed the trends. We also compared practice-level and demographic characteristics – specifically, physician sex and practice location - across four-year-long groupings using repeated-measures ANOVA, grouping by time period and individual physician IDs. Each unit of observation is an FP-year combination, containing data on the number of patient contacts per physician per fiscal year. As a result, FPs are observed repeatedly across the dataset, with each observation providing new information on that FP’s practice patterns at a given time in their career, although our aggregate-level descriptive approach does not specific analyse individual-level career trajectories. Since new FPs enter practice and later-career FPs leave practice due to retirement, we also allow observations to enter and leave the dataset over the observational period.

## Results

The total number of observations (for each fiscal year combination) with a general practice specialty was 110,884. After removing physicians with another specialty, fewer than 100 annual patient contacts, and missing data for sex or year of graduation, 106,199 observations remained, with a total of 9,709 unique FPs across the 21-year study period. Table 1 shows the included FPs, aggregated by ten-year-long graduation cohorts and four-year time periods (including one two-year time period for 2016/2017). This table shows that there were 28 FPs practicing across the 1996–1999 time period who graduated prior to 1947. For simplicity, we only refer to the first year of a given fiscal year (i.e., 1996 for 1996/1997).

**Table 1:** Number of FP observations practicing across each 4-Year time period, stratified by Graduation Cohort

	Graduation Year							
***Time Period***	**< 1947**	**1947–1956**	**1957–1966**	**1967–1976**	**1977–1986**	**1987–1996**	**1997–2006**	**2007–2017**
**1996–1999**	28	512	1,526	3,981	4,939	5,208	183	≤ 5
**2000–2003**	16	332	1,262	3,860	5.125	5,215	1,410	≤ 5
**2004–2007**	≤ 5	201	1,010	3,730	5,255	5,574	2,795	≤ 5
**2008–2011**	≤ 5	106	675	3,508	5,220	5,750	4,324	569
**2012–2015**	≤ 5	41	389	2,969	5,084	6,044	4,776	2,666
**2016–2017**	≤ 5	≤ 5	98	1,096	2,396	3,025	2,642	2,651

Table 2 presents the FPs in BC according to demographic and practice-related variables. Between 1996 and 2017, the number of FPs increased by nearly 50%. Overall, there were more male FPs than female FPs, although the percentage of females in the FP population increased from 29.8% in 1996–1999 to 42.3% in 2016–2017. The mean experience level of FPs grew in BC over this period, and the mean number of years in practice rose from 17.34 in 1996–1999 to 23.65 in 2016–2017 – reflecting the aging of the baby boomer generation. Similarly, the mean age of the FPs increased from 43.04 in 1996–1999 to 50.37 in 2016–2017. There was a small decline in the proportion of FPs practicing in urban locations, from 65.5% in 1996–1999 to 63.5% in 2016–2017.

**Table 2:** FP characteristics by Time Period

FP Demographics	1996-1999 (*N*=16,383)	2000-2003 (*N*=17,223)	2004-2007 (*N*=18,570)	2008-2011 (*N*=20,152)	2012-2015 (*N*=21,969)	2016-2017 (*N*=11,911)	F-Stat	*P* value
	*N* (%)	*N* (%)	*N* (%)	*N* (%)	*N* (%)	*N* (%)		
**Female**	4,887 (29.8%)	5,422 (31.5%)	6,246 (33.6%)	7,383 (36.6%)	8,811 (40.1%)	5,078 (42.6%)	4.56	<0.001
**Urban Practice**	10,738 (65.5%)	11,028 (64.0%)	11,842 (63.8%)	12,711 (63.1%)	14,073 (64.1%)	7,569 (63.5%)	2.13	0.087
**Smaller Urban Practice**	3,765 (23.0%)	3,961 (23.0%)	4,315 (23.2%)	4,808 (23.9%)	5,127 (23.3%)	2,817 (23.7%)	2.27	0.053
**Rural Practice**	1,880 (11.5%)	2,234 (13.0%)	2,413 (13.0%)	2,633 (13.1%)	2,769 (12.6%)	1,525 (12.8%)	2.87	0.011
	**Mean (SD)**	**Mean (SD)**	**Mean (SD)**	**Mean (SD)**	**Mean (SD)**	**Mean (SD)**		
**Years in Practice**	17.34 (10.93)	18.56 (10.99)	20.81 (11.45)	22.05 (11.29)	22.29 (12.91)	23.65 (13.13)	5.43	<0.001
**Age**	43.04 (10.7)	44.14 (10.75)	46.35 (11.01)	48.00 (10.91)	48.55 (12.38)	50.37 (12.98)	4.82	<0.001
**Annual Contacts**								
**Total Contacts**	4,847 (2,879)	4,791 (3,142)	4,759 (2,941)	4,694 (3,216)	3,799 (2,867)	3,547 (2,995)	2.63	0.003
**MHSU Contacts**	291 (456)	293 (520)	301 (529)	312 (620)	321 (922)	335 (856)	2.08	0.006
**MH Contacts**	243 (231)	225 (264)	236 (276)	245 (317)	207 (226)	205 (243)	2.94	0.012
**SU Contacts**	48 (378)	51 (412)	54 (487)	67 (517)	115 (876)	125 (865)	2.23	0.048

**Fig. 1:**
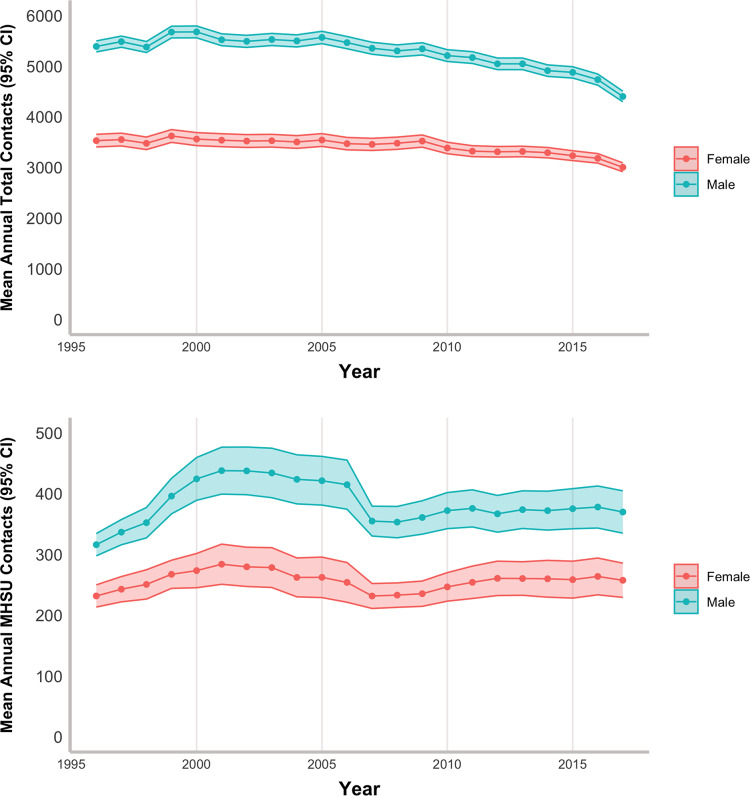
Mean annual contacts per physician across the time period, stratified by FP Sex

The mean number of total contacts per FP decreased by more than 20%, from 4,847 in 1996 to 3,799 in 2017. The number of annual MHSU contacts increased from 291 in 1996–1999 to 321 in 2016–2017. MH contacts decreased from 243 in 1996 to 207 in 2017, while SU contacts more than doubled from 48 in 1996 to 115 in 2017. Figure 1 and Tables S2 and S3 in Additional File 1 show these comparisons and highlight a prominent dip in MHSU contacts around the year 2007, particularly for male FPs. Tables S2 and S3 also show a more detailed breakdown of contacts by graduating cohort, graduation year, and other demographic variables. Figures S1 and S2 in Additional File 1 show specifically how MH and SU trends differ across the time periods – with mean MH contacts declining steadily across the time period, while mean SU contacts increased after an initial downward spike around 2007.

**Fig. 2:**
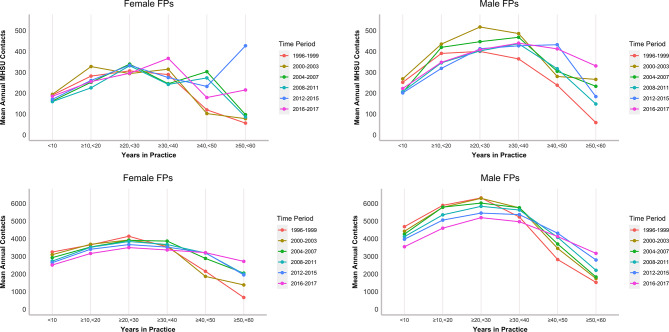
Mean annual contacts across age groups stratified by 4-year period

Figure 2 summarizes the mean annual contacts per FP by career stage group and four-year-long time period, with complete statistical results presented in Tables S4 and S5 in Additional File 1. Figure 2 shows that across all time periods, there is a clear relationship between years in practice and mean annual contacts: there is a clear upside-down U-shaped relationship as practice volume initially starts low, increases until mid-career, and then steadily decreases until retirement. This pattern is observed across all four-year-long time periods for both males and females and is evident when considering total and MHSU contacts, although the trend is less pronounced for MHSU contacts. Figure 2 shows that FPs in earlier time periods generally had greater service volume in the early part of their career but lower levels of service volume later in their career. This is shown most prominently in the total contacts. Over our period of analysis, the mean number of annual contacts (both total and MHSU) was lower for females than for males, although the effect of career stage was less pronounced for females than for males.

**Fig. 3:**
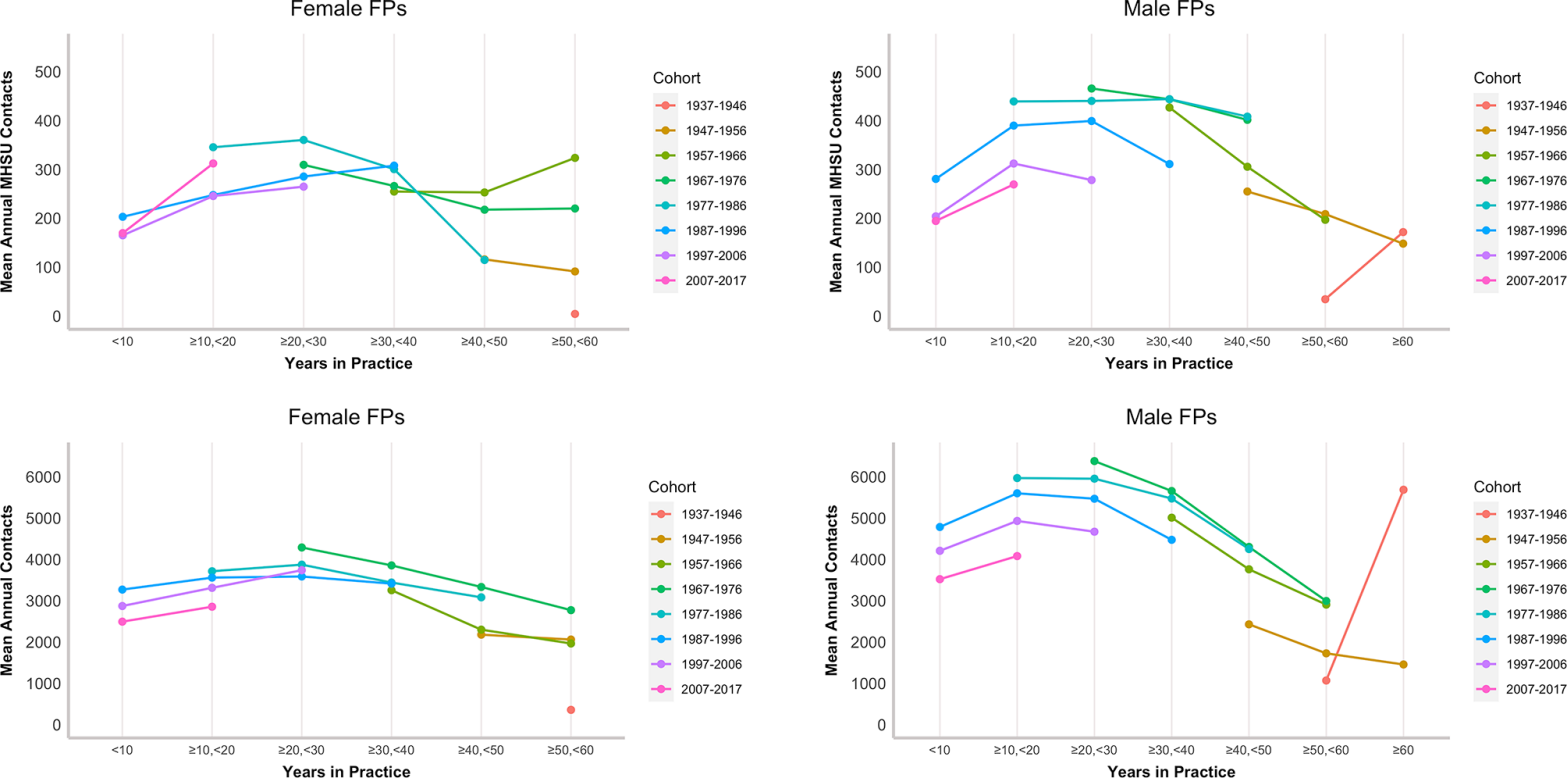
Mean annual total and MHSU contacts by cohort and age

Figure 3 shows the cohort trends for total and MHSU contacts, with the statistical results presented in Tables S6 and S7 in Additional File 1. Although there are no cohorts that are observed across the entirety of their careers, Fig. 3 points to the same inverse U career trend: a steady increase until mid-career, followed by a decline in late career. We can also see an interesting pattern among later career FPs: in some cohorts, practice volumes increased beyond 50–60 years in practice. A key finding from Fig. 3 is that more recent graduating cohorts had lower overall contact volume than earlier graduating cohorts at most career stages. This is evident for both males and females at early career stages. This trend is also observed for MHSU services but less so for female FPs. The opposite, however, is true among FPs with more than 30 years in practice, although the relationship is slightly less clear.

## Discussion

Our study explored how contact volume – and more specifically, MHSU contact volume – relates to demographic trends in the FP workforce in BC. We found that the number of FPs in BC increased by nearly 50% between 1996 and 2017, and the demographics of the workforce overall changed; the number and proportion of female FPs grew over this period, and the mean number of practice years increased by five years. These findings are consistent with previous research documenting the aging of the baby boomer generation of FPs [25, 30, 39], and the increasing proportion of female FPs in Canada [23] and BC more specifically [2].

Our study showed a more than 20% reduction in FP mean total contact volume from 1996 to 1999 to 2016–2017; this was driven by a combination of a 17% reduction among male FPs (from 5,481 to 4,567), 13% among female FPs (from 3,545 to 3,090), and an increase in the total proportion of female FPs (from 30 to 43% of the total population). We also find that recent graduates had lower contact volume than predecessors at the same career stages. However, this trend reverses in late career, where later graduation cohorts exhibit greater contact volumes than earlier graduation cohorts. This pattern could reflect a cohort effect, suggesting that recent graduates practice differently than predecessors at identical career stages, or it could also reflect a period effect, indicating that changes in practice context affect all cohorts concurrently.

Previous studies have not found evidence of cohort effects impacting FP practice [25, 30, 39]. Furthermore, our results show declines in service volume among both early- and mid-career FPs, which suggests that changes in practice are not unique to recent graduates. Other evidence proposes that FPs of all career stages are being impacted by changes in practice context, such as increasing complexity of care [42–44] and growing administrative burden [45, 46]. These demands could erode service volumes across all career stages. Further research employing more sophisticated modeling strategies is required to isolate the impacts of graduation cohort from other effects on the workforce.

While physicians’ overall contact volume decreased over the 21-year study period, MHSU contacts increased. This was largely driven by more than a doubling of visits related to substance use. The data also highlight a prominent dip in MHSU contacts around 2007 (specifically SU services, and particularly for male FPs), which coincides with an amendment to the billing code for methadone treatment in BC [47]. It is unclear why MHSU contacts did not decline with overall contact volume, but this could be related to increasing demand for community-based mental health services (although evidence suggests that these services continue to be underfunded) [48]. In addition, there is a divergence between MH and SU contacts; as MH contacts decreased, SU contacts increased. This suggests that any of the conclusions drawn here about how years in practice, generational cohort, or time period affects mean annual contacts may not apply uniformly to either MH or SU contacts. There are many potential reasons for this, including changes in patient complexity, administrative burden, and patient-level factors such as changes in literacy around MHSU treatment. There is also the possible influence of the change in methadone billing on service volume, so it is likely that there are some unique factors that are driving changes in these two categories separately. This motivates a need for further research into these two components separately, as there is a distinct need from the BC context for research into access to primary substance use services.

With respect to the FP’s career stage, we observed an inverse U-shaped trend, with physicians’ contact volume rising steadily from start to mid-career, peaking at 20–30 years in practice, and decreasing steadily thereafter. In several cohorts, FPs increased practice volume beyond 60 years of practice. This is driven by the small number of FPs who are likely outliers compared to their contemporaries who retired from clinical practice. These findings mirror those of Rudoler et al. (2022).

The FP workforce is also aging (from a mean age of 43 years in 1996 to a mean age of 50 in 2017), which will lead to a decline in supply as the peak of the FP age distribution shifts closer to retirement. The inverse U-shaped career trend also applies to MHSU contacts but peaks at a later career stage (at 30–40 years of practice) in the most recent study period. Some Canadian evidence points to education and preparedness playing a role in an FP’s level of comfort delivering mental health services [49]; however, additional research is needed to understand why FPs may provide more MHSU services later in their careers.

A career trend was apparent for both male and female FPs, although males had higher overall and MHSU volumes of contacts, as well as a steeper decline in contacts in late career. Norton et al. and Slade et al. reported fewer hours of work [50, 51] and fewer patient contacts with females than with males; however, female FPs spend more time with their patients and may be more likely to tackle multiple problems at one visit [23].

Based on our findings, two workforce trends confront policy makers as they consider the future supply of primary MHSU services: declining total service volume, declining MH service volume, and an aging workforce. Furthermore, if the demand for MHSU remains high and the supply of FP-delivered MHSU services remains steady—as has been over the last 20 years—it may start to crowd out the supply of other FP services. The workforce is also aging, which may exacerbate existing supply shortfalls as current mid-career physicians approach retirement. To combat these issues, an additional supply of FPs will be needed. The BC government has taken steps in their latest master agreement with Doctors of BC to increase the supply of FPs and their services by increasing compensation via an alternative payment model believed to be more conducive to comprehensive primary care [52]. However, there is no guarantee that this change will lead to increased supply, as an income effect may prompt some physicians to reduce their service volume [53–56].

Enticing new FPs is not the only means available to compensate for the dwindling supply of services. It also may not be effective given the trend of how recent entrants to the FP workforce have been observed to begin their practice at lower service volumes and attain fewer peaks during their mid-career. This phenomenon might be attributable to the escalating demands on FPs to undertake nonclinical tasks such as care coordination and laboratory testing, as well as an increase in the number of forms and EMRs required for patient referrals and diagnostic procedures which are competing for their time and resources [45, 46]. Providing support for primary care practices to reduce administrative burdens may help individuals increase their supply. Interprofessional team-based care remains as a potential solution for access shortfalls in the sector. This approach continues to provide the promise of improving access to comprehensive primary care services to the extent that it involves optimal use of all existing primary care human resources, i.e., expanded scope of practice. The promise of team-based care is particularly persuasive in MHSU service delivery where collaborative care models that integrate specialized mental health support into primary care are supported by compelling evidence [57, 58].

This study has several limitations. We focused only on contact volume, which prevents us from commenting on the quality of care. The study does not address patient-level factors that might influence the level of service provision, such as differences in population-level patient complexity or patient-level demand for MHSU services. There are other factors that influence service provision outside of the career stage, time period, and cohort effects that we did not explore, such as training location (national versus international) and differences in practice geography (rural vs. urban). The dataset includes clinical data only and therefore does not account for activities that FPs may be undertaking outside their clinical roles, such as administrative tasks, teaching, or other activities. Additionally, since the dataset does not provide detailed information on the amount of time that FPs spend with patients, we have no way of converting our measure of service provision to full-time equivalent (FTE) metrics, potentially limiting the comparability of the study. Regarding male/female FPs, it is not clear whether the sex variable captures legal sex, sex assignment at birth, or self-identified gender.

There are also several limitations related to the fact that there may have been important and distinct changes to the FP practice environment. It is unclear how our results interact with the care provided by other medical professionals and non-medical MHSU service providers. Patients facing complex care needs, for example, may be receiving care from psychiatrists either additional to or instead of care from FPs. As part of wider care networks, non-physician MHSU providers such as psychologists, psychotherapists, social workers, harm reduction workers, and peer support providers can play a significant role both in primary MHSU service provision [59, 60]. Psychiatric and non-physician practice patterns may have changed over this period, affecting the environment in which all FPs practice.

A key strength of the study is the richness of its data source, which allowed us to draw trends from nearly 10,000 FPs across two decades of billing data. Because the data are administrative rather than survey or self-reported billing data, there is high internal validity, with the potential for the study methods to be used among a variety of other, similar research settings.

There are several areas for future research stemming from our study, including an exploration into FPs with fewer than 100 patient contacts annually. This group represents around 5% of FPs in BC, and their exclusion from this analysis was due to the understanding that they may represent a very different approach to service provision compared to FPs with more active practices. Another opportunity is the exploration of the impact of the opioid crisis on the province. Between 1996 and 2014, the rate of illicit drug toxicity deaths per 100,000 people in BC remained below 400; however, from 2015 to 2017, it escalated dramatically from 529 to 1,493 per 100,000. [61] Our current findings do not indicate any discernible influence of the increased presence of fentanyl and sedatives in BC’s drug supply on the practice patterns of FPs. It is plausible that patients affected by drug-related harm may face barriers to accessing primary care, potentially limiting the observable impact on FP practices during this period. The duration of our data collection may not capture changes in primary health service utilization due to the crisis adequately. Additionally, our data does not extend to observing potential trends resulting from the COVID-19 pandemic. Exploring these aspects in future research could provide valuable insights into the broader implications of the opioid crisis on healthcare delivery and provider practices in BC.

## Conclusions

Our study underscores the pressing challenges facing policymakers in ensuring an adequate supply of primary MHSU services in BC. The dual trends of declining service volume for MH-specific and total contacts and an aging workforce present significant hurdles, potentially straining the overall provision of healthcare services. While the government’s efforts to increase the supply of FPs are noteworthy, it remains uncertain whether these changes will fully address the growing demand. Furthermore, the evolving dynamics of FP practice, including increasing administrative burden, necessitate a comprehensive approach that includes reducing administrative tasks and exploring the potential of interprofessional team-based care to enhance access and quality of care in primary care settings.

There is no informed consent required for this study because the data is from an administrative health services database. Our REB has been satisfied with the explanation we have provided which follows: “We are using administrative data and no consent is needed for use of this data. All data will be de-identified. For this research, all physicians that delivered care in one or more years between 1996/7 to 2016/17 and all of the contacts they had with patients during those years will be included.”

In accordance with Article 3.7 A of the Tri-Council Policy Statement: Ethical Conduct for Research Involving Humans, this study meets the conditions for alternations to consent requirements. As the research involves no more than minimal risk to the participants, the alteration of consent is unlikely to adversely affect the welfare of participants, and since all data will be de-identified, it will be impracticable to obtain informed consent. Sub-articles (d) and (e) of Article 3.7 A do not apply (Canadian Institutes of Health Research, Natural Sciences and Engineering Research Council of Canada, and Social Sciences and Humanities Research Council of Canada, Tri-Council Policy Statement: Ethical Conduct for Research Involving Humans, December 2022).

## Electronic supplementary material

Below is the link to the electronic supplementary material.

Supplementary Material 1

## Data Availability

The data that support the findings of this study are available from Population Data BC but restrictions apply to the availability of these data, which were used under license for the current study, and so are not publicly available. Access to data provided by the Data Stewards is subject to approval but can be requested for research projects through the Data Stewards or their designated service providers. The following data sets were used in this study: (Medical Services Plan Payment Information, CPSBC registry, consolidation files). You can find further information regarding these data sets by visiting the PopData project webpage at: https://my.popdata.bc.ca/project_listings/19-044/collection_approval_dates. All inferences, opinions, and conclusions drawn in this publication are those of the author(s), and do not reflect the opinions or policies of the Data Steward(s).

## Abbreviations

BC: British columbia
CPSBC: College of physicians and surgeons of BC
FP: Family physician
FTE: Full-time equivalent
ICD: International classification board
MHSU: Mental health and substance use
MSP: Medical services plan
SD: Standard deviation
