# Supplementary material for: Demographic and temporal trends in mental health and substance use services provided by primary care physicians in British Columbia, Canada
Source: BMC Prim Care. 2024 Sep 10;25:335. doi: 10.1186/s12875-024-02587-y (PMC11384687; doi:10.1186/s12875-024-02587-y)
Supplement: Supplementary file 1 — Supplementary Material 1 [file 12875_2024_2587_MOESM1_ESM.docx]

**Additional File 1**

*Table S1: ICD-9 codes used to identify mental health and substance use contacts, as described by Steele et al. (2004)*

| ***ICD-9 Codes*** | | ***Categorization as Mental Health or Substance Use*** |
| --- | --- | --- |
| ***Substance Use Disorders*** | 303 Alcoholism  304 Drug dependence | Substance Use |
| ***Psychotic Disorders*** | 95 Schizophrenia  296 Manic-depressive psychoses, involutional melancholia  297 Other paranoid states  298 Other psychoses | Mental Health |
| ***Non-Psychotic Disorders*** | 300 Anxiety neurosis, hysteria, neurasthenia, obsessive-compulsive neurosis, reactive depression  301 Personality disorders  302 Sexual deviations  306 Psychosomatic illness  309 Adjustment reaction  311 Depressive disorder  50B Anxiety/depression  04A General psychiatric examination, no care required | Mental Health |
| ***Social Problems*** | V60.2 Inadequate material resources  V61.1 Marital Problems  V61.2 Parent-child problems  V61.3 Problems with aged parents or in-laws  V61.0 Family disruption  V62.3 Educational circumstances  V62.4 Social maladjustment  V62.0 Unemployment  V62.1 Adverse effects of work environment  V62.5 Legal Circumstances  V62.8 Other psychological or physical strain, not elsewhere classified  V62.9 Unspecified other psychosocial circumstances | Mental Health |

*Table S2: Summary of Mean Yearly Contacts (rounded to nearest whole number)*

|  | ***FP Sex*** | | ***FP Location*** | | |
| --- | --- | --- | --- | --- | --- |
| ***Year*** | **Female** | **Male** | **Metro** | **Smaller Urban** | **Rural** |
| **1996-1999** | 3,545 | 5,481 | 4,995 | 5,062 | 4,066 |
| **2000-2003** | 3,537 | 5,553 | 5,162 | 4,915 | 3,719 |
| **2004-2007** | 3,492 | 5,471 | 4,064 | 4,782 | 3,575 |
| **2008-2011** | 3,423 | 5,255 | 4,872 | 4,493 | 3,359 |
| **2012-2015** | 3,287 | 4,970 | 4,514 | 4,313 | 3,146 |
| **2016-2017** | 3,090 | 4,567 | 4,176 | 3,883 | 2,854 |
| ***Years Since Graduation*** |  | | | | |
| **0-9** | 2,851 | 4,243 | 3,679 | 3,638 | 2,779 |
| **10-19** | 3,502 | 5,539 | 4,926 | 4,596 | 3,614 |
| **20-29** | 3,795 | 5,898 | 5,392 | 5,227 | 4,115 |
| **30-39** | 3,578 | 5,496 | 5,325 | 5,080 | 3,523 |
| **40-49** | 2,953 | 3,893 | 4,038 | 3,428 | 2,511 |
| **50-59** | 1,906 | 2,347 | 2,428 | 2,154 | 1,471 |
| **≥60** | 2,654 | 3,265 | 3,252 | n/a | 3,044 |
| ***Year of Graduation*** |  | | | | |
| **2007-2017** | 2,496 | 3,529 | 3,156 | 2,831 | 2,326 |
| **1997-2006** | 3,083 | 4,567 | 3,911 | 3,977 | 3,020 |
| **1987-1996** | 3,501 | 5,383 | 4,821 | 4,583 | 3,493 |
| **1977-1986** | 3,714 | 5,807 | 5,326 | 5,113 | 4,040 |
| **1967-1976** | 3,916 | 5,588 | 5,560 | 5,210 | 3,716 |
| **1957-1966** | 2,769 | 4,209 | 4,205 | 4,108 | 2,971 |
| $\boldsymbol{\leq}$**1956** | 2,137 | 2,198 | 2,025 | 3,093 | 2,142 |

*Table S3: Summary of Mean Yearly MHSU Contacts (rounded to nearest whole number)*

|  | ***FP Sex*** | | ***FP Location*** | | |
| --- | --- | --- | --- | --- | --- |
| ***Year*** | **Female** | **Male** | **Metro** | **Smaller Urban** | **Rural** |
| **1996-1999** | 249 | 350 | 344 | 311 | 197 |
| **2000-2003** | 279 | 433 | 422 | 380 | 210 |
| **2004-2007** | 252 | 404 | 386 | 349 | 193 |
| **2008-2011** | 243 | 366 | 353 | 306 | 191 |
| **2012-2015** | 260 | 372 | 357 | 327 | 174 |
| **2016-2017** | 261 | 374 | 354 | 325 | 188 |
| ***Years Since Graduation*** |  | | | | |
| **0-9** | 176 | 229 | 220 | 199 | 141 |
| **10-19** | 267 | 385 | 383 | 291 | 196 |
| **20-29** | 322 | 433 | 423 | 401 | 233 |
| **30-39** | 285 | 440 | 429 | 434 | 211 |
| **40-49** | 214 | 352 | 346 | 387 | 150 |
| **50-59** | 165 | 203 | 214 | 192 | 78 |
| **≥60** | 113 | 158 | 152 | n/a | 173 |
| ***Year of Graduation*** |  | | | | |
| **2007-2017** | 172 | 196 | 192 | 206 | 119 |
| **1997-2006** | 204 | 258 | 254 | 212 | 174 |
| **1987-1996** | 250 | 371 | 366 | 274 | 176 |
| **1977-1986** | 341 | 441 | 429 | 448 | 230 |
| **1967-1976** | 273 | 442 | 448 | 385 | 227 |
| **1957-1966** | 258 | 347 | 305 | 527 | 163 |
| $\boldsymbol{\leq}$**1956** | 107 | 239 | 231 | 287 | 79 |

*Table S4: Mean Total Contacts by 4-Year Groups and Years Since Graduation*

|  | ***Females*** | | | | | |
| --- | --- | --- | --- | --- | --- | --- |
|  | ***1996-1999*** | ***2000-2003*** | ***2004-2007*** | ***2008-2011*** | ***2012-2015*** | ***2016-2017*** |
| ***Years Since Graduation*** | Mean (SD) | Mean (SD) | Mean (SD) | Mean (SD) | Mean (SD) | Mean (SD) |
| **0-9** | 3,242 (2,226) | 3,090 (2,220)* | 2,291 (2,109)* | 2,724 (2,062)** | 2,636 (1,981) | 2,504 (1,935)* |
| **10-19** | 3,645 (2,004) | 3,679 (2,379) | 3,524 (2,511)* | 3,509 (2,545) | 3,399 (2,429) | 3,157 (2,281)** |
| **20-29** | 4,135 (2,461) | 3,924 (2,447) | 3,905 (2,509) | 3,836 (2,586) | 3,661 (2,526)* | 3,493 (2,349)* |
| **30-39** | 3,523 (2,546) | 3,616 (2,599) | 3,861 (2,888) | 3,666 (2,842) | 3,520 (2,649) | 3,358 (2,581) |
| **40-49** | 2,146 (1,985) | 1,858 (1,564) | 2,878 (2,443)* | 3,185 (2,588) | 3,197 (2,819) | 3,205 (3,052) |
| **50-59** | 663 (334) | 1,375 (1,661) | 2,045 (1,450) | 2,000 (1,257) | 1,944 (2,195) | 2,711 (2,342) |
|  | ***Males*** | | | | | |
|  | ***1996-1999*** | ***2000-2003*** | ***2004-2007*** | ***2008-2011*** | ***2012-2015*** | ***2016-2017*** |
| ***Years Since Graduation*** | Mean (SD) | Mean (SD) | Mean (SD) | Mean (SD) | Mean (SD) | Mean (SD) |
| **0-9** | 4,679 (3,014) | 4,418 (3,099)** | 4,255 (2,988) | 4,112 (2,958) | 3,968 (3,019) | 3,542 (2,641)*** |
| **10-19** | 5,887 (3,009) | 5,775 (3,267) | 5,785 (3,452) | 5,349 (3,214)*** | 5,051 (3,080)*** | 4,593 (3,113)*** |
| **20-29** | 6,309 (2,810) | 6,287 (3,228) | 6,016 (3,453)*** | 5,831 (3,440)* | 5,448 (3,330)*** | 5,189 (3,303)** |
| **30-39** | 5,227 (2,782) | 5,747 (3,100)*** | 3,753 (3,221) | 5,662 (3,490) | 5,363 (3,445)** | 4,959 (3,314)*** |
| **40-49** | 2,813 (2,355) | 3,445 (2,797)*** | 3,695 (2,962) | 4,072 (3,185)** | 4,304 (3,289) | 4,116 (3,138) |
| **50-59** | 1,518 (1,445) | 1,735 (1,733) | 1,825 (1,899) | 2,205 (2,300) | 2,789 (2,637) | 3,165 (2,851) |
| **≥60** |  | 05,247 (161 | 6,114 (523) |  | 1,001 (1,647) | 2,430 (1,597) |
| P-values reflect results from two-sampled mean comparison test between consecutive four-year groups. | | | | | | |
| *5% level, **1% level, ***.1% level | | | | | | |

*Table S5: Mean MHSU Contacts by 4-Year Groups and Years Since Graduation*

|  | ***Females*** | | | | | |
| --- | --- | --- | --- | --- | --- | --- |
|  | ***1996-1999*** | ***2000-2003*** | ***2004-2007*** | ***2008-2011*** | ***2012-2015*** | ***2016-2017*** |
| ***Years Since Graduation*** | Mean (SD) | Mean (SD) | Mean (SD) | Mean (SD) | Mean (SD) | Mean (SD) |
| **0-9** | 191 (288) | 194 (298) | 162 (202)*** | 159 (249) | 170 (515) | 185 (655) |
| **10-19** | 282 (474) | 327 (815)** | 252 (607)*** | 225 (371) | 261 (651)* | 255 (610) |
| **20-29** | 307 (357) | 294 (386) | 339 (861) | 331 (794) | 334 (866) | 295 (639) |
| **30-39** | 289 (437) | 315 (415) | 245 (261)** | 242 (240) | 275 (743) | 367 (1201)* |
| **40-49** | 120 (204) | 102 (126) | 303 (672)** | 273 (420) | 232 (335) | 179 (231)* |
| **50-59** | 56 (83) | 77 (102) | 96 (107) | 856 (77) | 427 (1,016) | 215 (295) |
|  | ***Males*** | | | | | |
|  | ***1996-1999*** | ***2000-2003*** | ***2004-2007*** | ***2008-2011*** | ***2012-2015*** | ***2016-2017*** |
| ***Years Since Graduation*** | Mean (SD) | Mean (SD) | Mean (SD) | Mean (SD) | Mean (SD) | Mean (SD) |
| **0-9** | 252 (484) | 269 (619) | 203 (360)*** | 207 (351) | 201 (371) | 222 (537) |
| **10-19** | 390 (730) | 435 (1,064)* | 420 (1,170) | 345 (806)** | 319 (761) | 348 (984) |
| **20-29** | 400 (600) | 517 (1,147)*** | 447 (1,044)** | 402 (768)* | 413 (972) | 408 (1,055) |
| **30-39** | 364 (771) | 486 (1,229)*** | 467 (1,115) | 437 (995) | 427 (962) | 440 (1,133) |
| **40-49** | 238 (631) | 280 (953) | 305 (1,027) | 317 (857) | 432 (1,253)** | 412 (1,272) |
| **50-59** | 59 (93) | 266 (1,066) | 233 (898) | 148 (352) | 183 (238) | 331 (1,004) |
| **≥60** |  | 160 (35) | 183 (27) |  | 50 (99) | 328 (312) |
| P-values reflect results from two-sampled mean comparison test between consecutive four-year groups. | | | | | | |
| *5% level, **1% level, ***.1% level | | | | | | |

*Table S6: Mean Yearly MHSU Contacts by Graduating Cohort and Years Since Graduation*

|  | ***Females*** | | | | | | | |
| --- | --- | --- | --- | --- | --- | --- | --- | --- |
|  | ***<1946*** | ***1947-1956*** | ***1957-1966*** | ***1967-1976*** | ***1977-1986*** | ***1987-1996*** | ***1997-2006*** | ***2007-2017*** |
| ***Years Since Graduation*** | Mean (SD) | Mean (SD) | Mean (SD) | Mean (SD) | Mean (SD) | Mean (SD) | Mean (SD) | Mean (SD) |
| **0-9** |  |  |  |  |  | 203 (317) | 165 (280)*** | 169 (564) |
| **10-19** |  |  |  |  | 345 (830) | 247 (483)*** | 245 (594) | 312 (968) |
| **20-29** |  |  |  | 309 (344) | 360 (930)** | 285 (577)*** | 265 (247) |  |
| **30-39** |  |  | 255 (376) | 266 (310) | 300 (899) | 308 (609) |  |  |
| **40-49** |  | 116 (190) | 253 (616)** | 217 (240) | 114 (90)*** |  |  |  |
| **50-59** | 4 (5) | 91 (93)*** | 323 (793) | 220 (310) |  |  |  |  |
|  | ***Males*** | | | | | | | |
|  | ***<1946*** | ***1947-1956*** | ***1957-1966*** | ***1967-1976*** | ***1977-1986*** | ***1987-1996*** | ***1996-2007*** | ***2007-2017*** |
| ***Years Since Graduation*** | Mean (SD) | Mean (SD) | Mean (SD) | Mean (SD) | Mean (SD) | Mean (SD) | Mean (SD) | Mean (SD) |
| **0-9** |  |  |  |  |  | 280 (600) | 204 (325)*** | 194 (430) |
| **10-19** |  |  |  |  | 439 (929) | 390 (997)** | 312 (833)*** | 269 (488) |
| **20-29** |  |  |  | 466 (906) | 440 (942) | 399 (968)** | 278 (332)** |  |
| **30-39** |  |  | 427 (1,181) | 443 (984) | 444 (1,080) | 311 (893) |  |  |
| **40-49** |  | 255 (802) | 305 (990) | 401 (1,172)*** | 408 (1,073) |  |  |  |
| **50-59** | 34 (47) | 208 (813) *** | 197 (432) | 569 (1,691) |  |  |  |  |
| **≥60** | 172 (31) | 148 (234) |  |  |  |  |  |  |
| P-values reflect results from two-sampled mean comparison test between consecutive graduating cohorts. | | | | | | | | |
| *5% level, **1% level, ***.1% level | | | | | | | | |

*Table S7: Mean Yearly Contacts by Graduating Cohort and Years Since Graduation*

|  | ***Females*** | | | | | | | |
| --- | --- | --- | --- | --- | --- | --- | --- | --- |
|  | ***<1946*** | ***1947-1956*** | ***1957-1966*** | ***1967-1976*** | ***1977-1986*** | ***1987-1996*** | ***1996-2007*** | ***2007-2017*** |
| ***Years Since Graduation*** | Mean (SD) | Mean (SD) | Mean (SD) | Mean (SD) | Mean (SD) | Mean (SD) | Mean (SD) | Mean (SD) |
| **0-9** |  |  |  |  |  | 3,265 (2,268) | 2,868 (2,101)*** | 2,489 (1,895) *** |
| **10-19** |  |  |  |  | 3,709 (2,089) | 3,555 (2,510)** | 3,307 (2,379)*** | 2,850 (2,008) |
| **20-29** |  |  |  | 4,284 (2,728) | 3,868 (2,504)*** | 3,582 (2,420)*** | 3,734 (2,328) |  |
| **30-39** |  |  | 3,250 (2,148) | 3,850 (3,058)*** | 3,436 (2,511)*** | 3,409 (2,036) |  |  |
| **40-49** |  | 2,175 (1,918) | 2,295 (2,082) | 3,328 (2,903)*** | 3,078 (2,657) |  |  |  |
| **50-59** | 355 (220) | 2,056 (1,439)*** | 1,961 (2,041) | 2,767 (2,089) |  |  |  |  |
|  | ***Males*** | | | | | | | |
|  | ***<1946*** | ***1947-1956*** | ***1957-1966*** | ***1967-1976*** | ***1977-1986*** | ***1987-1996*** | ***1996-2007*** | ***2007-2017*** |
| ***Years Since Graduation*** | Mean (SD) | Mean (SD) | Mean (SD) | Mean (SD) | Mean (SD) | Mean (SD) | Mean (SD) | Mean (SD) |
| **0-9** |  |  |  |  |  | 4,780 (3,083) | 4,203 (3,004)*** | 3,516 (2,691)*** |
| **10-19** |  |  |  |  | 5,963 (3,094) | 5,595 (3,342)*** | 4,926 (3,058)*** | 4,076 (2,978)* |
| **20-29** |  |  |  | 6,374 (2,919) | 5,948 (3,362)*** | 5,463 (3,408)*** | 4,665 (2,767)* |  |
| **30-39** |  |  | 5,005 (2,908) | 5,650 (3,192)*** | 5468 (3,552)** | 4,469 (3,084)** |  |  |
| **40-49** |  | 2,424 (2,243) | 3,756 (2,879)*** | 4,297 (3,273)*** | 4,246 (3,296) |  |  |  |
| **50-59** | 1,066 (1,180) | 1,723 (1,778)** | 2,901 (2,675)*** | 2,989 (2,832) |  |  |  |  |
| **≥60** | 5,680 (588) | 1,453 (1,611)*** |  |  |  |  |  |  |
| P-values reflect results from two-sampled mean comparison test between consecutive graduating cohorts. | | | | | | | | |
| *5% level, **1% level, ***.1% level | | | | | | | | |


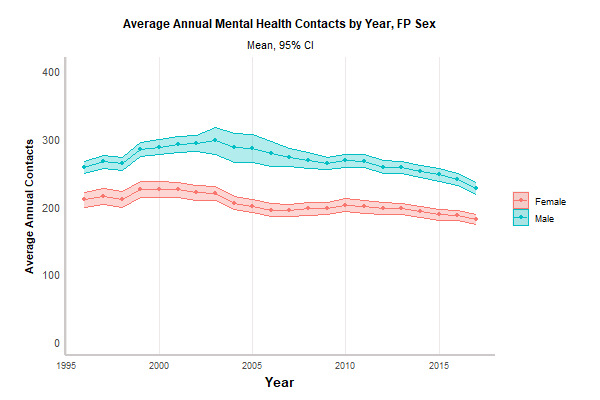


*Figure S1: Mean Yearly MH Contacts per Physician across the* *Time Period, Stratified by FP Sex*


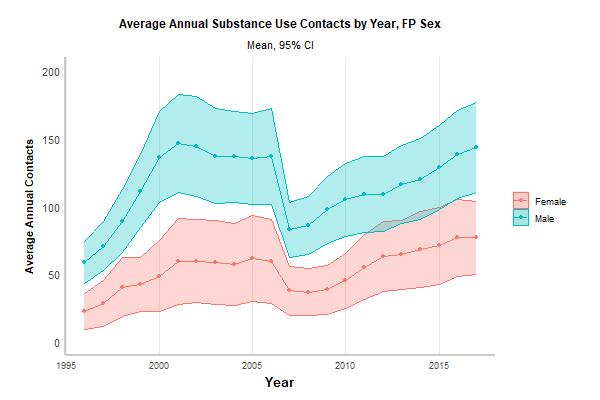


*Figure S2: Mean Yearly SU Contacts per Physician across the Time Period, Stratified by FP Sex*
